# Supplementary material for: Ubiquitin-related genes are differentially expressed in isogenic lines contrasting for pericarp cell size and grain weight in hexaploid wheat
Source: BMC Plant Biol. 2018 Jan 25;18:22. doi: 10.1186/s12870-018-1241-5 (PMC5784548; doi:10.1186/s12870-018-1241-5)
Supplement: Supplementary file 3 — Q-value distributions of uniquely DE transcripts across time. (DOCX 171 kb) [file 12870_2018_1241_MOESM3_ESM.docx]

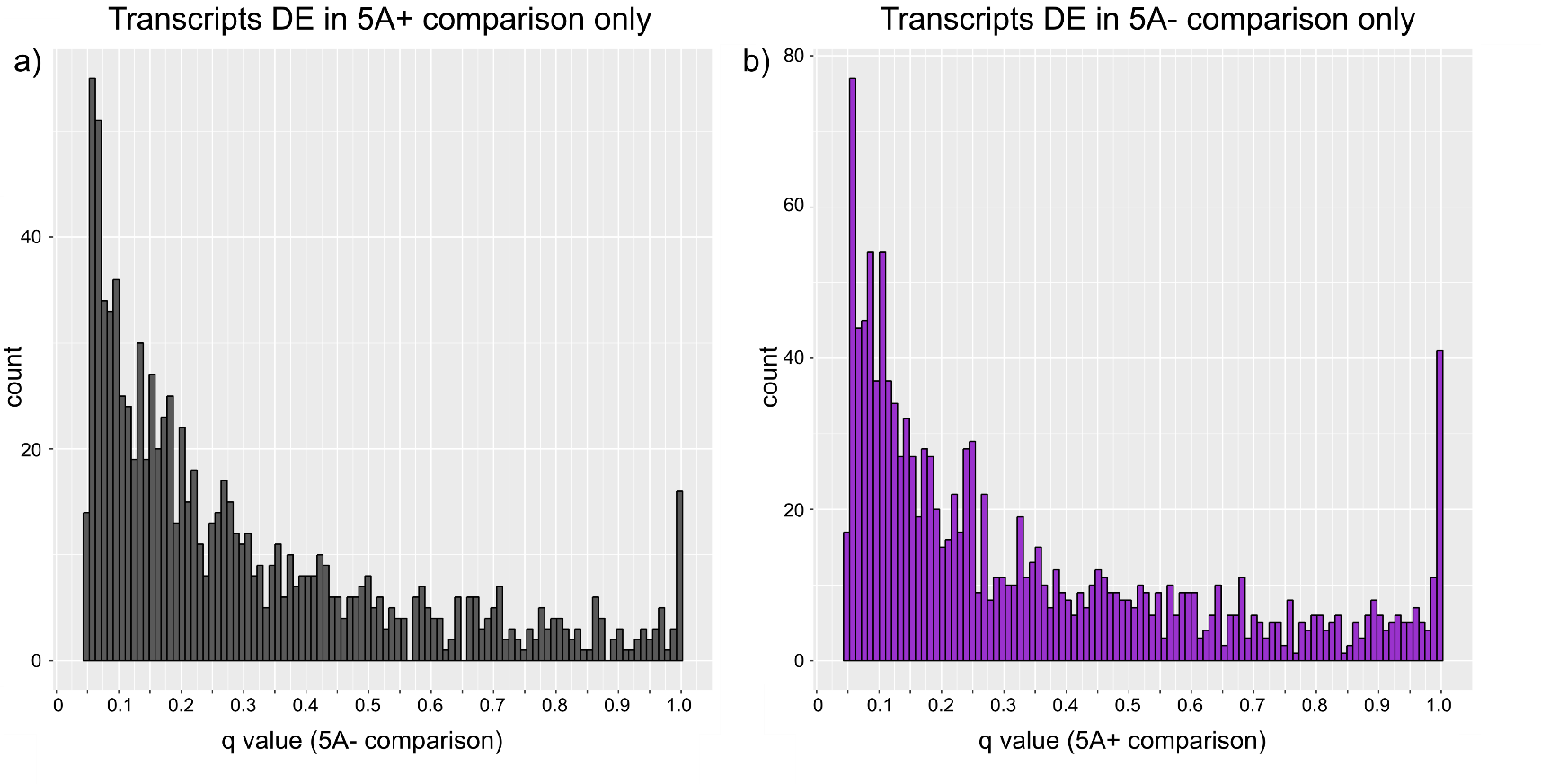


**Additional file 3: q-values distributions of uniquely differentially expressed transcripts across time**

a) Distribution of ${\text{5A-}\text{ }}_{\text{T2}}^{\text{T1}}$ q-values for transcripts that were differentially expressed (DE) only in the ${\text{5A+}\text{ }}_{\text{T2}}^{\text{T1}}$ comparison (and not the ${\text{5A-}\text{ }}_{\text{T2}}^{\text{T1}}$ comparison). b) Distribution of ${\text{5A-}\text{ }}_{\text{T2}}^{\text{T1}}$ q-values for DE transcripts across time in the ${\text{5A-}\text{ }}_{\text{T2}}^{\text{T1}}$ comparison only. The fact that both distributions are skewed towards lower q-values shows suggests that many of the DE genes within a single comparison were borderline non-significant in the opposite comparison.
