# Supplementary material for: Ubiquitin-related genes are differentially expressed in isogenic lines contrasting for pericarp cell size and grain weight in hexaploid wheat
Source: BMC Plant Biol. 2018 Jan 25;18:22. doi: 10.1186/s12870-018-1241-5 (PMC5784548; doi:10.1186/s12870-018-1241-5)
Supplement: Supplementary file 7 — qRT-PCR expression values and graphs. (DOCX 370 kb) [file 12870_2018_1241_MOESM7_ESM.docx]

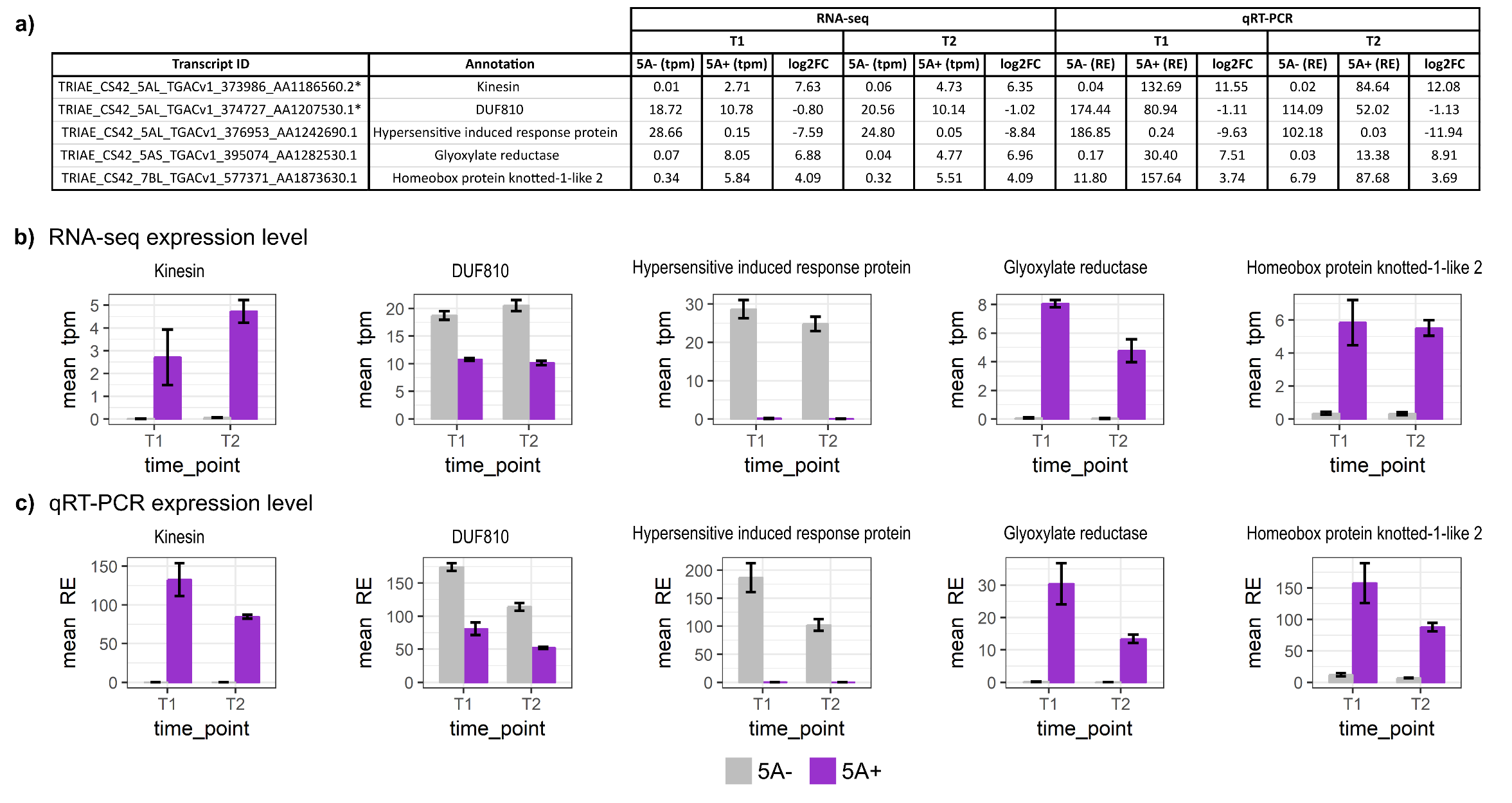


**Additional file 7: Quantitative reverse-transcript PCR (qRT-PCR) of a subset of differentially expressed (DE) transcripts**

a) Table of expression values for five DE transcripts obtained from RNA-seq data and qRT-PCR. tpm = transcripts per million, RE = relative expression to actin (10^-3^), log2FC = log2 fold change in expression in 5A+ near isogenic line (NIL) compared to 5A- NIL, * indicates transcripts located within the fine mapped region on chromosome 5A. b) Bar charts showing mean tpm of the five transcripts obtained using RNA-seq. c) Bar charts showing mean RE of the five transcripts obtained using qRT-PCR. In all cases error bars represent standard error of the mean, n = 3 biological replicates. Grey bars = 5A- NIL, purple bars = 5A+ NIL. All pairwise comparisons between NILs are significantly different as determined by t-tests at both timepoints 1 and 2.
